# Paediatric otogenic tetanus: an evidence of poor immunization in Nigeria

**DOI:** 10.11604/pamj.2017.26.177.11519

**Published:** 2017-03-29

**Authors:** Segun Ayodeji Ogunkeyede, Adekunle Daniel, Omowonuola Ogundoyin

**Affiliations:** 1 Department of Otorhinolaryngology, College of Medicine, University of Ibadan, Ibadan and University College Hospital, Ibadan Nigeria

**Keywords:** Childhood, Nigeria, otogenic tetanus, paediatric, tetanus immunization

## Abstract

Suppurative otitis media is a common childhood infection that predisposes to otogenic tetanus. Tetanus is a vaccine preventable disease that is associated with high cost of care and mortality. This study highlights reasons for otogenic tetanus in Nigerian children and way of reducing the menace. This is a 5-year retrospective review of all patients managed for otogenic tetanus in at the Department of Otorhinolaryngology, University College Hospital, Ibadan. The data collected include demographic, clinical presentations, tetanus immunisation history, and duration of hospital admission, and management- outcome. There were 23 patients comprising of 13(56.5 %) males and 10 (43.5%) females, male to female ratio was 1.3:1. The age ranged between 11 months and12 years (mean age 3.4 years ± 2.1). All the patients presented with discharging ear, trismus and spasms. The onset of symptoms prior hospital presentation ranged between 2 - 11 days (mean 3.0 days ± 1.3). Only 12(52.1%) patients had complete childhood tetanus immunisation, 6(26.1) % had no tetanus immunisation and no other childhood immunisation, while 5(21.7%) had partial tetanus immunisation. The discharging ears were managed by self-medication and other harmful health practices. The hospital admission ranged from 20 days - 41days (average of 23days) and there were 3(13.0 %) death. Tetanus immunization was not received because of; non- availability of the vaccine at health centers, lack of health facility in communities, fear of complications from immunization, poor awareness of the immunization programme. Tetanus, an immunisable disease, is still a major problem in Nigeria.

## Introduction

In spite of simple preventive measures available through immunization, tetanus remains a major cause of mortality in a resource challenged environment, like Nigeria. Tetanus spores are introduced into the body by attitudes/ practices that expose open wounds to contamination from soil or animal faeces [1]. Insertion of contaminated object picked from the bare floor to clean the discharging ear is a possible source of infection. The exotoxins (Tetanospasmin) produced by tetanus organisms initiate a cascade in the nervous system that leads to the clinical manifestations which include locked jaw, dysphagia, muscular rigidity and Spasm . Otitis media is inflammation of the middle ear cleft. It is a common childhood infection, affecting diverse racial and cultural groups, predominantly in the developing countries with limited resources. The burden of the disease entity is enormous, especially in Sub-Saharan region of the African continent, with its attendant economic implications [2, 3].

Immunization is an important public health intervention and constitutes a cost effective strategy to reduce both the morbidity and mortality associated with infectious diseases. immunization prevents infectious diseases, but in Nigeria, the immunization programme suffers recurrent setbacks due to many factors, and vaccine preventable deaths accounts for nearly twenty percent of childhood deaths [4]. Shortage of vaccines and immunization supplies has always been problematic in Nigeria, primarily because funds are not sufficient and are not timely released [5]. This study evaluated otogenic tetanus among the paediatric patients managed for chronic suppurative otitis media and factors that predispose to otogenic tetanus.

## Methods

This is a retrospective study of paediatric patients diagnosed with otogenic tetanus managed in the department of Otorhinolaryngology, University College Hospital, Ibadan Nigeria, between September 2011 and October 2016. The hospital records, operation register and admission records were retrieved. The data extracted included demographic data (age, sex), the maternal educational status , clinical presentations, duration of symptoms, duration of stay in the hospital, mode of treatment of the discharging ear prior hospital presentation and in the hospital, tetanus immunization history, factors influencing tetanus immunization, and management outcome. All cases were diagnosed by using the clinical features of the disease. Socioeconomic status of the patients was based on parental occupational strata [6]. All the data were entered into the IBM-SPSS version 20.0 computer software for descriptive analysis and results are presented in tables.

## Results

There were 23 subjects made up of 13(56.5 %) males and 10 (43.4%) females. Theage ranged between 11 months and 12 years, mean of 3.4 years ± 2.1. All the patients were from low socioeconomic status, they all presented with ear discharge, trismus and spasms. The onset of symptoms prior hospital presentation ranged between 2 days and 11 days with a mean of 3.0 days ± 1.3. Only 12(52.1%) patients had complete childhood tetanus immunization,6(26.1%) had no tetanus immunization and other childhood immunizations, while 5(21.7%) had partial tetanus immunization, as shown in Table 1. Prior to hospital presentation all the mothers had used topical ear antibiotics and 86.9% had history of use of herbs, honey, cigarette filter, palm oil, coconut oil or hydrogen peroxide in the discharging ear, as shown in Table 2. While 9(39.1%) had consulted traditional healers, spiritual houses and local medicines shops at onset of tetanus symptoms. The parents were from low socioeconomic status and poor educational level; 18(78.3%) had primary school certificate and 5(21.7%) had no formal education, as shown in Table 3. All the patients were managed with topical antibiotic ear dressing, tetanus toxoid, human tetanus immunoglobulin, antibiotic therapy and sedatives. Ventilatory support was given to 3(13.0%) patients that were initially managed in the intensive care unit. The duration of hospital admission ranged from 20 days - 41 days (average of 23days) and there were 3(13.0 %) deaths. The cost of hospital care was high due to prolonged hospital admission. Factors identified for partial immunization or no immunization were non- availability of the vaccine at health centers in 5(21.7%), lack of health facility in the community in 3(13.0%), 2(8.6%) had fear of complications that might arise from been immunized, and 1(4.3%) of the parents was not aware of the immunization programme, as shown in Table 4.

**Table 1:** the patient’s immunisation status

Gender	Fully immunised	Partially immunised	Not immunised	Total
Male	7(30.4%)	1 (4.3%)	5(21.8%)	13(56.5%)
Female	5(21.8%)	4(17.4%)	1(4.3%)	10(43.5%)
**Total**	12(52.2%)	5(21.7%)	6(26.1) %	23(100%)

**Table 2:** materials used by the mothers in dressing the discharging ear

Materials applied into the discharging ear by mothers	Topical antibiotics	23(100%)
	Herbs	17(73.9%)
	Honey	10(43.5%)
	Cigarette filter	5(27.3%)
	Hydrogen peroxide	2(8.7%)
	Palm oil	4(17.4%)
	Coconut oil	1(4.3%)

Some patients used more than one item

**Table 3:** the educational level of the parents and the facilities where care was received at onset of tetanus symptoms before hospital presentation

Facility where care was received	Primary school education	No formal education	Total
Traditional healers,	1(11.1%)	1(11.1%)	2(22.2%)
Spiritual houses	1(11.1%)	2(22.2%)	3(33.4%)
Local medicines shops	2(22.2%)	2(22.2%)	4(44.4%)
Total	4(44.4%)	5(55.5%)	9(100%)

**Table 4:** factors limiting the rate of immunisation

Factors affecting immunisation	Affected patients
Lack of vaccine at health centers	5(45.4%)
Lack of health post in the community	3(27.3%)
Fear of complications that might arise from immunisation	2 (18.2%)
Lack of awareness of the immunisation programme	1(9.1%)
Total	11(100%)

## Discussion

Benefits of immunization include good health, cost-saving benefit through lowering the incidence of disease, and less frequent visits to the hospital, thus promoting survival of children. It also affords the parents the opportunity to maximally use their time and money. In this study the non- vaccination of the patients may be due to none awareness of the importance of immunization. Report has shown a low demand for immunization at the community level due to a lack of understanding of its value [7]. An active public campaign on significance of childhood immunization is needed in order to create the awareness of immunization, and for the populace to recognise the importance of immunization especially tetanus immunization which is part of the children’s routine immunization.

The non-availability of health institutions in some communities is a great barrier to tetanus immunization and basic primary health care. The lack of basic public health facilities in many communities in Nigeria has been reported in a previous study as a limitation to the immunization programme in Nigeria [8]. There is a need for a government policy on health matters that will strengthen the existing and provide health care centers in the localities with non–existing public health institutions. This can be achieved by collaborations with donor agencies, non-governmental organisations, private sectors and health professional bodies both internationally and locally.

It has been reported that high level of illiteracy is associated with low participation in immunization programme [8]. This may be responsible for non-immunization or partial tetanus immunization in this study, because all parents of these patients had low educational level. Mass education of the populace should be embarked upon by the government in order to reduce the level of illiteracy, and in the interest of the national development. The public should be educated on tetanus immunization and other routine immunization. This will make the mothers have confidence in immunization; because the misconception that immunization is a panacea for all childhood sicknesses reduces confidence in immunization as a mean of prevention for any diseases, when immunization fails to do such [9].

The fear of complications arising from immunization that prevented some mothers from immunizing their children as seen in this study is a reflection of rumors and religious bias against immunization in some parts of Nigeria. Previous report shows that community leaders and religious leaders who are decision-makers have rejected routine immunization due to incorrect information, and this rejection had a negative impact on the parents’ decision on their children’s immunization [10]. The government should incorporate the community and religious leaders into the decision making bodies on the immunization and other government policies on health, in order for them to understand the importance of immunization, and influence the populace positively.

The non-availability of vaccines at health post as reported by the mothers at time of visit for routine immunization, is a reflection of the shortage of vaccines and immunization supplies in Nigeria. This is due to insufficient and delayed funds which prevented procurement of the vaccines or inadequate supply of vaccines [5, 11]. This might also be due to non-availability, poorly equipped, ill-managed facilities, and/or under-utilisation of the storage facilities for the vaccines in those health posts to maintain the cold chain [12]. Poor and erratic electric-power supply in Nigeria may also hamper the storage facilities in those health posts, contributing to non-availability of the vaccines. The break in the cold chain might be responsible for those who had had full tetanus immunization but still went on to develop otogenic tetanus. The manufacturing companies of these vaccines should improve on technology for vaccine production, in such a way that vaccines can be stored at room temperatures in resource challenged countries where cold chain might not be sustainable. The united nations children`s fund (UNICEF) information on immunization in Nigeria shows that 47% of children now get the vaccination that protects them against diphtheria, tetanus and pertussis (DPT), while the proportion of children who are fully immunized in Nigeria in the past two decades has never really reached optimal levels. Current coverage rates for the various childhood vaccines in Nigeria are amongst the lowest in the world [10]. There is a need for the Nigeria government to have a review of the health policy on immunization in such a way that the logistics for vaccines procurement, storage and distribution to the point of usage will be borne by government and health insurance companies. This will reduce the government spending and improve the immunization coverage and adequate the monitoring.

All the mothers did not present at the hospital at the onset of the ear discharge, but practiced self-medication at home by applying antibiotic ear drops, other materials used to care for the discharging ear like cigarette filters, honey, charcoals, and herbs were risk factors for the otogenic tetanus. In spite of the tetanus symptoms, some parents delayed in seeking medical care. Initial care was at spiritual houses and traditional/ herb doctors as well as medicine stores in their locality. They presented in hospital when the symptoms persisted. Health education through mass media, at immunization and antenatal clinics on the need for tetanus immunization, avoidance of harmful practices and prompt hospital visit is needed, in order to educate the populace on the danger of self-medication, the harmful practices, delay in hospital presentation and preventive measures for otogenic tetanus. All the patients in this study had daily ear toileting and dressing with topical antibiotic based on the organism sensitivity pattern. None of the patients had tympanomastoid surgeries for the chronic suppurative otitis media, this was due to financial constrains (inability to afford radiological investigations and surgical fees for mastoid surgery). The health care should be made affordable to the populace by the government through the health policy on health insurance scheme.

## Conclusion

Otogenic tetanus is still a major problem in Nigeria. Health education on tetanus immunization and affordable health care for the populaces will reduce the burden of the disease. The primary health care policy in Nigeria should be strengthened.

### What is known about this topic

- Otogenic tetanus is associated with mortality and morbidity;
- Tetanus is preventable by vaccine (routine childhood immunization).

### What this study adds

- Harmful health practices is a risk factor for tetanus infection in suppurative otitis media;
- Poor level of immunization among the patients was partly due to non-availability of the vaccine at health post, due to poor planning and implementation of immunization programme;
- Poor awareness of importance of immunization and wrong belief debar parents from immunizing their children.
